# Burn care: before the burn center

**DOI:** 10.1186/s13049-020-00792-z

**Published:** 2020-10-02

**Authors:** David J. Dries

**Affiliations:** 1 Department of Surgery, HealthPartners Medical Group, St. Paul, USA; 2 Department of Surgery, University of Minnesota, Mpls, USA; 3 Regions Hospital, 640 Jackson Street, #11503C, St. Paul, MN 55101 USA

Many burn care papers and textbooks begin with presentation of the patient to the Emergency Department. Maudet and coworkers from Switzerland depart in an important way from this pattern by providing a 10 year review of pre-hospital Helicopter Emergency Medical Service (HEMS) management of burn patients in a homogeneous practice serving one of two Burn Centers in Switzerland [1]. Notably, in this with system, every EMS service automatically refers to the Burn Center. Thus, these authors have a good sample of the pattern for initial hospital management and prehospital care. Sophisticated pain protocols utilize fentanyl and ketamine. There is remarkable consistency in evaluation of burn size. Resuscitation complications are not identified. Consideration of this elegant work begins an important conversation in which I will present a North American perspective. My observations reflect experience with the extensive area of the North Central United States served by a network of small *safety net* hospitals providing care for a wide variety of problems and having inconsistent burn experience.

Burn injuries may be challenging to manage, and many hospitals scattered over a wide area do not have the personnel, resources, and expertise to care for these individuals. Consequently, the *American Burn Association*, in conjunction with the A*merican College of Surgeons Committee on Trauma* has developed referral criteria to help providers determine that patients should be transferred to a Burn Center (below) [2]. Many of these criteria relate to location, mechanism, or severity of the burn. In addition, there are criteria that recommend the transfer of specific groups of patients, such as children or patients with significant comorbidities or rehabilitation needs.

**Table:** 

Burn Center Referral Criteria	
**- Partial thickness burns > 10% TBSA**	
**- Burns to face, hands, feet, perineum, joints**	
**- Third-degree burns**	
**- Electrocution including lightning**	
**- Inhalation injury**	
**- Chemical burns**	
**- Pediatric burns**	
**- Burns with comorbid medical conditions or coincident trauma**	

Modified: Resources for Optimal Care of the Injured Patient, 2014 [2].

## Transfer

Air transport is heavily used in rural areas or by hospitals that are far from Burn Centers. Although this mode of transportation is safe for patients, it should be used judiciously. Air transport is expensive and available resources may support only a portion of transportation costs [3]. Moreover, some patients who are brought to the Burn Center by air are discharged within 24 h if their injuries are discovered to be minor [4]. The need for air transport was examined in a recent study. A particular focus in this report was air transport resulting from overtriage, which was defined as discharge shortly after being brought to the Burn Center.

The study population in this recent triage review consisted of 1331 patients transferred by air and admitted to a single regional Burn Center between January 2003 and June 2013 [4]. There were 256 patients in the entire overtriaged group because they were discharged within 24 h (***note:*** in the first 24 h, 38 patients died). The remainder of these patients were assigned to the accurately triaged group because they were hospitalized for more than 24 h. Comparing the groups, the accurately triaged patient had a higher mean total body surface area (TBSA) burned (15% vs 3.3%), a higher percentage of patients with partial-thickness burns greater than 10% TBSA (44.6% vs 2.3%) and a greater percentage of patients with 3rd degree burns (26% vs 7%) than the overtriaged patients. Within the overtriaged group, 236 patients were transferred from other medical facilities and these individuals were classified as secondary overtriage.

Given the frequency of air transport overtriage in this study, the authors conclude that for patients with minor burns, in whom injury severity and urgency for treatment is low, air transport may not be necessary because of high cost associated with patient transfer and the lack of change in management of patients [3]. Reasons for overtriage were suggested including over estimation of burn size by referring facilities, lack of experience and resources to care for burn patients, particularly children, unfamiliarity with managing less common etiologies of burns (chemical and electrical), and concern for airway compromise with possible smoke inhalation injury [4–6]. The authors conceded that all of the patients in this study population met at least one of the standard referral criteria listed. Proposed solutions to reduce the rate of air transport overtriage for patients with minor burns include emphasis on other modes of transportation such as ground ambulance or private vehicles, telemedicine, improved communication between referring facilities and Burn Centers, and *revised referral criteria*.

## Burn size evaluation

Maudet and coworkers, in the present report, demonstrate impressive agreement in burn size estimates before and after arrival at the Burn Center [1]. Unfortunately, practice in other regions does not replicate these results. Retrospective data from multiple North American Burn Centers suggest that many patients referred for large injuries were actually discharged within 24 h. Up to 70% of patients referred with overestimated burn size were discharged within 48 h. Only 30% of referred patients required surgery. Many referred patients were more likely to have appropriate triage to a follow-up clinic rather than receiving direct transfer to the Burn Center [7]. International Burn Centers have reported similar experiences. One study from Australia showed that in children, 33% of total referrals had not correctly estimated TBSA injury, 25% to referrals did not meet transfer criteria, and up to 40% of transfers were not indicated [8]. A study in the United Kingdom noted that 37% of transferred patient had inaccurate estimated TBSA injuries such that transfer may not have been indicated. When multiple studies were reviewed, on average, 45 to 77% of small burns (less than 20% TBSA) and 26 to 37% of larger burns could be managed without a specialized Burn Center [9].

Given the significant risk of overestimation of burn size, patients with relatively minor injuries who present to nonburn institutions often undergo unnecessary interventions and receive excessive amounts of fluid. The issue of excessive resuscitation is relevant in light of changes in the Advanced Burn Life Support (ABLS) approach (below) which have decreased the amount of fluid administered relative to TBSA burned. Growing evidence suggests that utilization of computer-based imaging technology is a more reliable and reproducible means to estimate burn size. Using computer technology, now available on mobile devices such as tablets, as a component of a telemedicine system, burn triage may be further enhanced [7, 10]. Many burn providers suggest that a more robust telemedicine system may bring about a shift in referral-based burn care that will enhance the ability of initial providers to provide early management of the highest quality.

## Resuscitation

The goal of burn resuscitation is to maintain adequate tissue perfusion and organ function while avoiding complications of over or under resuscitation. Fluid administration is managed on a continuous basis to provide optimal outcomes. The administration of excessive volumes of resuscitation fluid magnifies edema formation, leading to various types of resuscitation-related morbidity including extremity, orbital, and abdominal compartment syndromes along with pulmonary and cerebral edema [11]. Shock and organ failure, most commonly manifest by acute kidney injury, may occur as a consequence of hypovolemia in the patient with an extensive burn who is untreated or receives inadequate amounts of fluid. The increase in capillary permeability caused by burn injury begins in the immediate post-burn period, and reduction in effective blood volume is the most rapid at that time. Prompt administration of adequate amounts of resuscitation fluid is essential to prevent decompensated burn shock and organ failure. Delay in initiating resuscitation often leads to higher subsequent fluid requirements. Inhalation injury has also been associated with increased fluid requirements [12]. It is essential that resuscitation commence as close to the time of injury as possible. Crystalloids, in reduced amounts as described below, remain essential to initial resuscitation of burn patients.

The latest statement regarding initial fluid rates reflected in current content from the ABLS curriculum is particularly relevant to providers caring for burn-injured patients before arrival at the Burn Center. In prehospital and initial hospital care settings, before formal calculation of percent TBSA burned is available, fluid administration guidelines are based on patient age with a fixed initial fluid rate as a starting point as follows:

- 5 years and younger: 125 mL lactated Ringer’s solution per hour
- 6–13 years of age: 250 mL lactated Ringer’s solution per hour
- 14 years and older: 500 mL lactated Ringer’s solution per hour

This approach is reasonable in light of the reported inconsistency in burn size estimation prior to arrival at a Burn Center [13]. As patient weight in kilograms is obtained and percent of second and third degree burns is carefully determined, the ABLS 2018 fluid resuscitation calculations may be used to determine an adjusted fluid rate.

Inpatient resuscitation strategies are derived from two commonly applied resuscitation formulas: the Parkland formula (4 ml/kg/%TBSA burned/first 24 h) and the modified Brooke formula (2 ml/kg/%TBSA burned/first 24 h) [11]. For these traditional formulas, it is estimated that half of the calculated 24 h fluid volume should be administered within the first 8 h after burn injury with the second half of the calculated 24 h resuscitation volume provided over the subsequent 16 h in the first post-burn day. Fluid administration is titrated based on urinary output and other end organ function. Recent reports indicate that resuscitation based on the historic 4 mL lactated Ringer’s solution per kilogram per percent TBSA burn commonly results in over resuscitation. Thus, a modified Brooke formula is commonly employed.

If initial formal inpatient resuscitation is delayed, the first half of the resuscitation volume is completed over the number of hours remaining in the first 8 h after burn injury. If inpatient resuscitation is delayed beyond 6 h after burn injury, a Burn Center should be consulted for the most appropriate *“catch up”* fluid administration strategy. In general, administration of crystalloid fluids via bolus administration should be avoided unless the patient is hemodynamically unstable. Special cases such as the adult with high-voltage electrical injury with evidence of myoglobinuria as reflected by pigment in the urine or in children where the body surface area per unit body mass is greater will require relatively larger amounts of resuscitation fluid. The body surface area/body mass relationship of a child also suggests a smaller intravascular volume per unit of surface area burned making the child with burns more susceptible to fluid overload and hemodilution. Infants and young children should receive lactated Ringer’s solution with 5% dextrose at a maintenance rate in addition to burn resuscitation as described earlier. The addition of 5% dextrose is caused by an increased risk of hypoglycemia because of limited glycogen stores in infants and children.

To *“wrap up”*, clean dry dressings with protection from hypothermia are the only necessary prehospital wound care [13]. Short-acting medications in divided doses, as demonstrated by Maudet and coworkers, may be given intravenously to provide rapid pain relief and reduce hemodynamic changes [1].
